# Quality of life of coronary artery disease patients after the implementation of planning strategies for medication adherence^1^

**DOI:** 10.1590/0104-1169.0144.2519

**Published:** 2015

**Authors:** Laura Bacelar de Araujo Lourenço, Roberta Cunha Matheus Rodrigues, Thaís Moreira São-João, Maria Cecilia Gallani, Marilia Estevam Cornélio

**Affiliations:** 2 RN, MSc, Hospital do Coração de Campinas, Campinas, SP, Brazil; 3 PhD, Associate Professor, Faculdade de Enfermagem, Universidade Estadual de Campinas, Campinas, SP, Brazil; 4 Post-doctoral fellow, Faculdade de Enfermagem, Universidade Estadual de Campinas, Campinas, SP, Brazil; 5 PhD, Full Professor, Faculté des sciences infirmières, Université Laval, Québec, Canada; 6 PhD, Associate Professor, Universidade Federal de São Carlos, São Carlos, SP, Brasil

**Keywords:** Nursing, Medication Adherence, Health Behavior, Planning Techniques, Coronary Disease

## Abstract

**OBJECTIVE:**

to compare the general and specific health-related quality of life (HRQoL) between the Intervention (IG) and Control (CG) groups of coronary artery disease patients after the implementation of Action Planning and Coping Planning strategies for medication adherence and to verify the relationship between adherence and HRQoL.

**METHOD:**

this was a controlled and randomized study.

**RESULTS:**

the sample (n=115) was randomized into two groups, IG (n=59) and CG (n=56). Measures of medication adherence and general and specific HRQoL were obtained in the baseline and after two months of monitoring.

**CONCLUSION:**

the findings showed that the combination of intervention strategies - Action Planning and Coping Planning for medication adherence did not affect the HRQoL of coronary artery disease patients in outpatient monitoring.

## Introduction

Patient adherence to medication therapy is essential for the control of coronary artery disease (CAD) and prevention of its complications^(^ 1 ^)^, constituting one of the biggest challenges for the nursing care to coronary disease patients, given the high percentage of non-adherence to the therapeutic medication regimen^(^ 2 ^)^. This construct refers to the extent to which patients follow the instructions of their physician or other healthcare professionals^(^ 3 ^)^. It is a complex phenomenon influenced by a range of factors, including, individual beliefs, skills, financial resources and/or barriers, and social influences^(^ 3 ^)^. Among the causes of non-adherence, the need for continued treatment can be highlighted, as well as the perceived lack of immediate benefits, the potential for adverse effects, and the costs associated with the treatment^(^ 3 ^)^.

Medication adherence can have significant impact on the health-related quality of life (HRQoL) in patients with chronic clinical conditions^(^ 4 ^-^ 5 ^)^, including CAD^(^ 6 ^)^. Although they are different constructs, adherence and HRQoL are related to patients and should be considered when evaluating the impact of interventions that affect their health^(^ 7 ^)^_._ Furthermore, these constructs are considered distinct outcomes in the care process - while adherence constitutes an intermediate result, HRQoL can be understood as a final outcome of the treatment^(^ 7 ^)^. Thus, it is possible to assume that interventions outlined for optimizing adherence influence medication adherence *a priori* and subsequently the HRQoL.

Although targeted at different health conditions, some studies have investigated the relationship between HRQoL and adherence. Among these, cross-sectional studies with geriatric hypertensive patients^(^ 5 ^)^, the use of medication for treatment of acquired immunodeficiency syndrome^(^ 8 ^)^ and treatment with lipid-lowering drugs^(^ 9 ^)^ can be highlighted. A recent literature review showed that few longitudinal studies have evaluated the impact of a theory-based intervention for the promotion or optimization of medication adherence on the general and specific HRQoL of patients with chronic^(^ 10 ^)^ clinical conditions, especially among CAD patients.

Considering the importance of medication adherence and HRQoL in CAD secondary prevention programs, this study aimed to compare the general and specific HRQoL measures of coronary artery disease patients randomized into the intervention group (IG) and control group (CG) before and after the implementation of Action Planning and Coping Planning strategies for medication adherence, and to verify the relationship between medication adherence and general and specific HRQoL throughout the monitoring.

## Methods

### Subjects and Procedures

Data from this study are derived from a broader experimental study^(^ 11 ^)^ that evaluated the effect of planning strategies on medication adherence and HRQoL of coronary artery disease patients. The sample consisted of CAD patients aged over 18, with prior clinical manifestation of angina or myocardial infarction (MI), with more than six months since the last ischemic event, and undergoing outpatient monitoring at one of the two hospitals in the state of São Paulo. Those who demonstrated an ability to communicate verbally and had been continuously using at least two oral drugs for CAD treatment (cardio protective and symptom relief medications) for at least a month were included. Those whose pharmacological treatment has been suspended or modified at the time of the initial approach (T_0_) were excluded. The participation of those patients who did not attend the scheduled follow-up appointments at one (T_1_) and two months (T_2_) after the baseline was discontinued, as well as those whose pharmacological therapy was suspended or modified during the monitoring period.

### Sample

After the acceptance of the invitation to participate in the study and provision of the formal consent by signing the Terms of Informed Consent, the subjects were randomized into control group (CG) or intervention group (IG). The aleatorization was based on a random sequence list generated by the SAS, version 9.1.3 program (SAS Institute Inc., Cary NC, USA, 2002-2003). The patients of the IG were submitted to the planning strategies - Action Planning and Coping Planning, applied by the main researcher; while the patients in the CG received usual care.

### Data Collection

Data were gathered from June 2010 to May 2011, using structured interviews and consultation of the hospital medical records at two different times:

- T_0_ (baseline): interview and consent of the patient to participate in the study obtained by signing the term. Data related to sociodemographic and clinical characterization, medication adherence^(^ 12 ^)^, factors related to non-adherence^(^ 13 ^)^ and general and specific^(^ 15 ^)^ HRQoL^(^ 14 ^)^ were obtained;

- T_2 _(two months after T_0_): adherence and HRQoL measures were again obtained.

### Intervention

The intervention was applied at T_0_ with only those patients in the IG and consisted of the formulation and implementation of plans, according to the theoretical assumptions of Implementation Intention^(^ 16 ^-^ 17 ^)^, based on a previous Brazilian study^(^ 18 ^)^. Patients were asked to design, in conjunction with the researcher, action and coping plans related to medication adherence. At T_1_ (one month after T_0_) presential reinforcement of the planning strategies was performed, by reading together the plans prepared at T_0._ The details of the intervention can be found in a previous study^(^ 11 ^)^.

### Control

The patients allocated to the CG received the routine care of the unit, which consisted of the usual clinical monitoring performed at the outpatient clinic. They were instructed to maintain their routine activities as well as their clinical follow-up appointments with the physician.

### Instruments

*- *The Morisky Self-Reported Measure of Medication Adherence Scale^(^ 13 ^)^: composed of four questions relating to non-adherence to pharmacological treatment, structured in Likert-type scales with four or five answer choices, the sum of which generates a score ranging from 4 up to 18; the lower the score the higher the favorability to adherence.

*- *Proportion of medication adherence (*Proporção de adesão medicamentosa*)^(^ 12 ^)^: constructed to identify and quantify medications and their form of use. It includes the following variables: Name, strength and dosage of the prescribed medications; Description of the form of use of each medication, according to the strength and dosage, in the previous 24 hours; in the previous week, and in the month prior to the interview. Adherence was calculated based on self-reported missed doses, using the calculation: [(Prescribed doses - missed doses) x 100 / prescribed doses]^(^ 19 ^)^. Those who obtained a percentage of consumption of prescribed medications, equal to or greater than 80%^(20) ^were considered "Adherent". For those who used more than one medication, the proportion of adherence was calculated through the mean of the percentages of adherence to each medication^(^ 12 ^)^. The proportion of adherence was analyzed as a continuous and binary variable - *appropriate dose* (dose used ≥80% of the prescribed dose) and *inappropriate dose* (dose <80% of the prescribed dose).

*- *Overall adherence assessment: the number and frequency of medications taken were evaluated, as well as their association with temporal markers: fasting, breakfast, lunch and dinner. This was evaluated based on the following classification: Group I (appropriate dosage and care for the prescription); Group II (appropriate dosage and inappropriate care), Group III (inappropriate dosage and appropriate care) and Group IV (inappropriate dosage and care). The patients classified in group I were considered "Adherent", and those classified in groups II, III, and IV "Non-adherent" ^(^ 12 ^)^. 

*- *The MacNew Heart Disease Health-related Quality of Life Questionnaire (MacNew): consists of a modified version of the "Quality of Life after Myocardial Infarction" (QLMI) original^(^ 21 ^)^ instrument and is composed of the domains: Physical Functioning (13 items), Emotional Functioning (14 items) and Social Functioning (13 items). An item can be part of more than one domain. The maximum possible score in any domain is seven (indicating the best HRQoL) and the minimum is one (suggesting the worst HRQoL). Items not answered do not contribute to the score and item 27 (sexual relations) can be excluded without affecting the final score of the domain. Domain scores are calculated through the arithmetic mean of the responses of that domain. If more than 50% of the items of a domain are not answered, the score for that domain is not calculated. The total score is calculated through the arithmetic mean of all the items answered, unless one of the domains is completely missing^(^ 22 ^)^. The Brazilian version of MacNew^(^ 15 ^)^ is considered reliable, valid and simple to apply^(^ 15 ^,^ 23 ^)^. In the present study the reliability with respect to the internal consistency, assessed through 

Cronbach's alpha coefficient, ranged between 0.80 and 0.90 throughout the monitoring.

*- *The 36-item Short Form Health Survey - SF-36: is a generic evaluation instrument of the perceived health status^(^ 24 ^)^, which is easy to administer and comprehend. It consists of eight domains: Functional Capacity (ten items), Physical Aspects (four items), Pain (two items), General Health Status (five items), Vitality (four items), Social Aspects (two items), Emotional Aspects (three items), Mental Health (five items), and one question comparing the current health conditions with those of one year previous. The final score ranges from zero (worst health status) up to 100 (best health status)^(^ 24 ^)^_._ The Brazilian version of the SF-36^(^ 14 ^)^ was used and, in the present study, the internal consistency, assessed through Cronbach's alpha coefficient, ranged between 0.80 and 0.90 throughout the monitoring.

### Data Analysis

Descriptive analyzes were performed to characterize the sample according to sociodemographic, clinical, medication adherence, and HRQoL variables. Student's t-test was used to check for differences between sociodemographic and clinical variables and the general and specific HRQoL between the IG and CG groups at T_0_. The paired Student's t-test was used to check for differences in the HRQoL means between the pre- (T_0_) and post-intervention (T_2_) times. Simple linear regression analyzes were used to evaluate change in the HRQoL at T_2_. Pearson's correlation coefficient was used to verify the relationship between medication adherence and HRQoL. Correlation coefficients < 0.30 were considered of weak magnitude, between 0.30 and 0.50 as moderate and > 0.50 of strong magnitude^(^ 25 ^)^. The significance level adopted for the statistical tests was p ≤ 0.05.

### Ethical Aspects

The study was approved by the Research Ethics Committee of a university in the state of São Paulo (Document No. 802/2009) and the Research Ethics Committee of a municipal hospital in the state of São Paulo (Document No. 001-11), as determined by Resolution No. 196/96 of the National Health Council/Ministry of Health. Patients were invited to participate in the study through the explanation and careful reading of the consent form, being informed about the aims of the study and data collection procedure, as well as the voluntary nature of their participation, their guaranteed anonymity and freedom to withdraw at any time without any loss regarding healthcare.

## Results

### Sociodemographic and clinical Characterization

At T_0_, 134 patients were considered eligible for the study. Of these, 8 were excluded due to the presence of at least one exclusion criterion. Thus, the sample consisted of 126 patients, of whom 62 were allocated to the CG and 64 to the IG. After the first approach, 5 patients were excluded from the CG and 6 from the IG due to non-attendance of the scheduled appointments. Thus, 115 patients completed the study (CG= 56; IG= 59). The comparative analysis between the groups at T_0_ showed that there were no differences between the groups regarding the sociodemographic and clinical variables, except for the number of previous myocardial infarctions, which was significantly higher in the IG (p=0.02).

The majority of the subjects consumed a mean of 6.4 (2.0) types of medication, with a mean of 3.6 (0.6) cardioprotective medications, and 0.8 (0.7) symptom relief medications. When using the proportion of adherence and the Morisky adherence scale, it was observed that the group was characterized by non-adherence. When considering the proportion of adherence in association with appropriate care, the majority was classified as non-adherent. However, in the IG there was a significant percentage of individuals that were adherent, when using the overall evaluation of adherence measure (p=0.023) (Table 1).

**Table 1 -:** Sociodemographic and clinical characterization of the Total 115 patients with Coronary Artery Disease and of the patients in the Intervention Group (n=59) and Control Group (n=56) in the baseline (T0). Campinas, SP, Brazil, 2010-2011

Variables			Total Group (n=115)	Intervention Group (N=59)	Control Group (N=56)
Sociodemographic					
	Age, Mean (SD*)		62.0 (9.0)	63.4 (8.9)	60.6 (9.0)
	Gender, n (%)				
		Male	75 (65.2)	40 (67.8)	35 (62.5)
	Marital Arrangement, n (%)				
		With partner	83 (72.2)	44 (74.6)	39 (69.6)
	Education (years), Mean (SD*)		5.8 (4.1)	5.5 (4.0)	6.2 (4.3)
	Habits/Lifestyle, n (%)				
		Current smoker (yes)	13 (11.3)	3 (5.1)	10 (17.8)
	Employment Situation, n (%)				
		Unemployed	71 (61.7)	44 (74.6)	33 (58.9)
		Employed	31 (26.9)	9 (15.2)	22 (39.3)
		Housewife/husband	10 (8.7)	5 (8.5)	5 (8.9)
	Family income (MW^†^), Mean (SD*)		3.0 (3.6)	3.2 (3.6)	2.7 (3.5)
Clinical					
	Characterization of coronary disease, n (%)				
		Unstable Angina	24 (20.9)	15 (25.4)	9 (16.1)
		Myocardial infarction	91 (79.1)	44 (74.5)	47 (83.9)
	Number of previous infarctions, Mean (SD*)		0.7 (1.1)	1.0 (1.3)^‡^	0.5 (0.9)^‡^
	Symptoms (in the previous month)^§^, n (%)				
		Precordialgia	51 (44.3)	31 (52.5)	20 (35.7)
		Dyspnoea	48 (41.7)	24 (40.7)	24 (42.8)
		Lower limb edema	40 (34.8)	23 (39.0)	17 (30.3)
		Palpitations	32 (27.8)	18 (30.5)	14 (25.0)
		Lipothymy	27 (23.5)	16 (27.1)	11 (19.6)
	Number of Associated Clinical Conditions, Mean (SD*)		5.0 (1.8)	5.1 (1.9)	4.8 (1.8)
	Associated Clinical Conditions^§^, n (%)				
		Dyslipidemia	91 (79.1)	51 (86.4)	40 (71.4)
		Arterial Hypertension	88 (76.5)	50 (84.7)	38 (67.8)
		Diabetes mellitus	41 (35.6)	27 (45.8)	14 (25.0)
	Treatment, n (%)				
		Clinical and Intervention (MR^||^/MRA^¶^/MR^||^ and MRA^¶^)	84 (73.0)	40 (67.4)	44 (78.6)
		Clinical	31 (27.0)	19 (32.2)	12 (21.4)
	Number of medications in use, Mean (SD*)		6.4 (2.0)	6.8 (2.0)	5.9 (1.9)
	Number of Cardioprotective Medications, Mean (SD*)		3.6 (0.6)	3.5 (0.6)	3.7 (0.5)
	Number of Symptom Relief Medications, Mean (SD*)		0.8 (0.7)	0.9 (0.7)	0.8(0.8)
	Total Morisky score, Mean (SD*)		6.9 (2.8)	7.3 (3.1)	6.5 (2.3)
	Proportion of Adherence, Mean (SD*)		92.9 (12.1)	92.1 (13.7)	93.7 (10.2)
	Proportion of Adherence^§^, n (%)				
		Appropriate dose (≥80%)	53 (89.8)	52 (88.1)	50 (89.3)
		Inappropriate dose (<80%)	6 (10.2)	7 (11.9)	6 (10.7)
	Overall adherence assessment^§^, n (%)				
		Adherent	27 (23.5)	19 (32.2)	8 (14.3)
		Non-Adherent	88 (76.5)	40 (67.8)	48 (85.7)

* Standard Deviation

† Minimum wage (R$ 545.00)

‡ Student's t test, p=0.02 §Percentage per line

|| Surgical Myocardial Revascularization

¶ Myocardial Revascularization by Angioplasty

### Analysis of Health Related Quality of Life (HRQoL) measures

Regarding the specific HRQoL (MacNew), significantly higher mean scores (p<0.05) for all domains of the MacNew were observed in the IG at T_2_, when compared to the baseline (T_0_). However, a significant increase in the scores of the majority of the MacNew domains was also observed in the CG, except in the emotional functioning domain, however, these differences were not statistically significant (Table 2). Regarding the generic HRQoL, significantly higher scores at T_2_ were observed in the IG in the Functional Capacity (p<0.001) and Emotional Aspects (p<0.05) domains, when compared to the scores obtained at T_0_. In the CG no significant differences were observed between the scores obtained at T_2_ and T_0_.

**Table 2 -:** Descriptive analysis of the scores of general (SF-36) and specific (MacNew) Health Related Quality of Life of coronary disease patients distributed into intervention group (n=59) and control group (n=62), at T0 and T2. Campinas, SP, Brazil, 2010-2011

		Intervention Group (n=59)				Control Group (n=56)		
		T_0_	T_2_	Mean differences (T_2_-T_0_)		T_0_	T_2_	Mean differences (T_2_-T_0_)
		Mean (SD*)	Mean (SD*)			Mean (SD*)	Mean (SD*)	
MacNew								
	Physical Functioning	4.8 (1.2)	5.2 (1.1)	0.4^†^		5.0 (1.3)	5.3 (1.1)	0.3^‡^
	Emotional Functioning	4.9 (1.1)	5.2 (0.9)	0.3^†^		5.2 (1.3)	5.4 (1.1)	0.2
	Social Functioning	4.7 (1.2)	5.1 (1.1)	0.4^†^		5.0 (1.2)	5.3 (1.1)	0.3^†^
	Total	4.8 (1.0)	5.1 (0.9)	0.3^†^		5.0 (1.1)	5.3 (1.0)	0.3^†^
SF-36								
	Functional Capacity	49.1 (22.3)	54.4 (28.7)	5.3^†^		55.4 (28.4)	58.4 (27.8)	3.0
	Physical Aspects	39.0 (29.1)	36.9 (38.1)	-2.1		43.7 (40.5)	44.2 (39.3)	0.5
	Pain	55.8 (24.3)	61.2 (25.7)	5.4		56.5 (28.0)	58.5 (27.2)	2.0
	General Health Status	59.0 (20.6)	61.1 (17.4)	2.1		60.2 (21.6)	57.4 (18.7)	-2.8
	Vitality	56.2 (23.2)	56.3 (21.1)	0.1		56.4 (22.9)	54.3 (21.9)	-2.1
	Social Aspects	60.4 (28.0)	70.8 (25.9)	10.4^†^		64.3 (29.6)	69.4 (26.6)	5.1
	Emotional Aspects	42.4 (39.1)	51.4 (41.2)	9.0		49.4 (41.2)	55.9 (38.2)	6.5
	Mental Health	61.4 (22.3)	62.4 (19.6)	1.0		63.1 (22.2)	62.6 (21.0)	-0.5

* Standard Deviation

† p<0.05

‡ p<0.001 - paired t-test

While the intervention explained 5% of the variability of the proportion of adherence measure^(^ 11 ^)^, the linear regression analysis showed that the Intervention was not able to explain the variability of the general and specific HRQoL measures.

### Relationship between Adherence and Health Related Quality of Life 

Regarding the correlations between the Morisky scale scores and those of the specific HRQoL, the absence of correlations was found in the IG, at T_0_. However, at T_2_, low magnitude negative correlations were observed with the Physical Functioning (r=-0.29; p=0.04), Emotional Functioning (r=-0.27; p=0.04) and Total Score (r=-0.29; p=0.04) domains of the MacNew, indicating that the higher the specific HRQoL, the greater the medication adherence. In the CG, at T_0_, a significant moderate magnitude negative correlation was found between the Morisky adherence measure and Emotional Functioning domain of the MacNew (r=-0.31; p=0.02). At T_2_, no correlations were found between the Morisky adherence scale and the specific HRQoL measure.

In the IG, at T_0_, no correlations were found between the Morisky adherence scale and the generic HRQoL. At T_2_ a weak positive correlation was found between the total score of the Morisky scale and the Emotional Aspects domain (r=0.27; p=0.04) of the SF-36, contrary to the previously established hypotheses. In the CG, at T_0_, weak negative correlations were found between the Morisky scale and the Pain (r=-0.26; p=0.05) and Mental Health (r=-0.29; p=0.03) domains, indicating that the better generic HRQoL the higher the favorability for adherence. At T_2_ no significant correlations were found for this group.

In the IG at T_0,_ no correlations were found between the proportion of adherence and the specific HRQoL. At T_2_, significant weak to moderate correlations were observed between the proportion of adherence and the Social Functioning domain (r=0.34; p=0.01) and Total Score (r=0.29; p=0.04) of the MacNew, indicating that the higher the specific HRQoL the better the adherence. However, in the CG at T_0,_ a negative correlation was found between the proportion of adherence and the Social Aspect domain (r= -0.28; p=0.04) of the MacNew, contradicting the previously formulated hypotheses. No significant correlations were found at T_2._

With regard to the relationship between the proportion of adherence and the general HRQoL measure, in the IG at T_0,_ a weak correlation was found with the General Health Status domain (r=0.26; p=0.05) of the SF-36. At T_2_ a moderate positive correlation was observed between the Emotional Aspects domain (r=0.30; p=0.02) of the SF-36, confirming the previously established hypotheses. However, in the CG at T_0_, significant low to moderate negative correlations were found between the proportion of adherence and the General Health Status (r= -0.42; p=0.00) and Social Functioning (r=- 0.28; p=0.04) domains of the SF-36, contrary to the previously established hypotheses. At T_2_ a significant moderate negative correlation was also found between the proportion of adherence and the General Health Status domain of the SF-36 (r=-0.28; p=0.03).

## Discussion

This study aimed to compare the general and specific HRQoL of coronary artery disease patients allocated in IG and CG after implementing an intervention based on Action Planning and Coping Planning strategies for medication adherence, as well as to verify the existence of a relationship between adherence and HRQoL over two months of monitoring.

The findings indicate that at T_2_, the IG patients presented a significant increase in mean scores in all the MacNew domains as well as the Functional Capacity and Emotional Aspects domains of the SF-36 when compared to the scores obtained at the start of the study (T_0_). Weak to moderate associations were found between adherence and the specific measure of HRQoL. However, the findings were not consistent over the subsequent two months.

The relationship between adherence and specific HRQoL (MacNew) were found especially in the IG at the end of the two month monitoring (T_2_), for both measures employed - proportion of adherence and the Morisky scale. Significant low magnitude negative correlations were found between the Morisky scale and the Physical Functioning, Emotional Functioning domains and the Total Score of the MacNew and weak to moderate correlations between the proportion of adherence and the Social Functioning domain and Total Score of the MacNew. However, an unexpected correlation in the CG at T_0_ was observed between the proportion of adherence and the Social Functioning domain of the MacNew. In summary, the greater the favorability of adherence, the better the HRQoL in all domains and the Total Score of the MacNew.

In the analysis of the relationship between adherence and general HRQoL (SF-36) the correlations occurred at both times (T_0_ and T_2_) and in both groups (IG and CG). In the IG the relationship was highlighted between the proportion of adherence and the General Health Status (at T_0_) and Emotional Aspects (at T_2_) domains. In the IG, the Morisky measure of adherence did not correlate with the general HRQoL, except for an unexpected positive correlation with the Emotional Aspect domain. In the CG negative correlations were found between the Morisky adherence scale and the Pain and Mental Health domains, while the proportion of adherence correlated negatively and unexpectedly with the General Health Status (at T_0_ and T_2_) and Social Aspects (at T_0_) domains. These findings suggest that the relationship between adherence and general HRQoL is less consistent compared to the results of the specific measure.

In the present study, although the IG presented better HRQoL in all domains of the MacNew and in the Functional Capacity and Emotional State domains of the SF-36, the intervention was not able to explain the variability in HRQoL after two months of monitoring. One explanation for this finding is the limitation of self-reported measures in the accurate measurement of adherence, as well as the limited time period for applying the intervention to change behavior as complex as adherence.

The weak to moderate magnitude correlations between adherence and HRQoL reported in the literature and demonstrated in the present study are consistent with the current recognition that other factors, in addition to the physical and emotional, affect the HRQoL. The exact mechanism by which medication adherence is associated with HRQoL is still unknown, with suggestions that HRQoL is part of a complex network of psychosocial characteristics that influence the patient's ability to cope with the chronicity of the disease^(5) ^.

Previous findings^(4) ^involving type 2 diabetic patients showed that medication adherence was not associated with the HRQoL domains. However, an association was observed with the combination of knowledge about the medical prescription and the attitude toward medication adherence, indicating the need for research into the determinant psychosocial variables for adherence behavior.

The absence or weak relationship between adherence and HRQoL were observed in other studies using self-reported measures^(^ 6 ^)^ as well as those using electronic records of prescriptions^(^ 4 ^,^ 8 ^)^_._ Thus, our results may reflect the limitations of different methods used for measuring medication adherence. Another limitation is the fact that, in the sample studied, the determinant psychosocial variables for medication adherence that could lead to the better design of the intervention were unknown - whether motivational and/or motivational and volitional. Further studies are recommended in order to deepen the knowledge about the longitudinal relationship between adherence to cardioprotective and symptom relief medications and HRQoL. Further investigation into the possible mediating effect of motivational and volitional determinants in medication adherence is suggested. The elucidation of these relationships will contribute to the design of theory-based interventions that are most effective in promoting adherence and, consequently, in improving HRQoL among CAD patients.

## Conclusion

The findings indicate that the intervention based on Action Planning and Coping Planning strategies for medication adherence did not affect the generic and specific measures of HRQoL in coronary disease outpatients. Regarding the relationship between adherence and HRQoL, significant, although weak to moderate, correlations were found between the medication adherence measures used in this study and the HRQoL measures, especially between medication adherence and the specific measure of HRQoL. Future studies are recommended in order to elucidate the psychosocial mediatory factors of the relationship between medication adherence and HRQoL, with a view to outlining nursing interventions effective in promoting medication adherence and improved HRQoL in patients suffering from coronary artery disease.

## References

[1] American Heart Association (2012). Heart disease and stroke statistics - 2012 Update: A
report from the American Heart Association. Circulation..

[2] Albert NM (2008). Improving medication adherence in chronic cardiovascular
disease. Crit Care Nurse..

[3] Haynes RB, Yao X, Degani A, Kripalani S, Garg A, McDonald HP (2005). Interventions for enhancing medication
adherence. Cochrane Database Syst Rev..

[4] Billups SJ, Malone DC, Carter BL (2000). The relationship between drug therapy noncompliance and
patients characteristics, health-related quality of life and health care
costs. Pharmacotherapy..

[5] Holt EW, Muntner P, Joyce CJ, Webber L, Krousel-Wood MA (2010). Health-related quality of life and antihypertensive
medication adherence among older adults. Age Ageing..

[6] Turpin RS, Simmons JB, Lew JF, Alexander CM, Dupee MA, Kavanagh P et al (2004). Improving treatment regimen adherence in coronary heart
disease by targeting patient types. Dis Manage Health Outcomes.

[7] Cotê I, Farris K, Feeny D (2003). Is adherence to drug treatment correlated with
health-related quality of life?. Qual Life Res..

[8] Holzemer WL, Corless IB, Nokes KM, Turner JG, Brown MA, Powell-Cope GM (1999). Predictors of self-reported adherence in persons living
with HIV disease. AIDS Patient Care STDS..

[9] Sung JC, Nichol MB, Venturini F, Bailey KL, McCombs JS (1998). Factors affecting patient compliance with anti
hyperlipidemic medications in an HMO population. Am J Manag Care..

[10] Williams A, Manias E, Walker R (2008). Interventions to improve medication adherence in people
with multiple conditions: a systematic review. J Adv Nurs..

[11] Lourenço LB, Rodrigues RC, Ciol MA, São-João TM, Cornélio ME, Dantas RA (2013). A randomized controlled trial of the effectiveness of
planning strategies in the adherence to medication for coronary artery
disease. J Adv Nurs..

[12] Jannuzzi FF (2009). Qualidade de vida relacionada à função visual e adesão
medicamentosa em idosos com retinopatia diabética. Dissertação [Mestrado].

[13] Ferreira MCS, Gallani MCBJ (2006). Adaptação transcultural do instrumento de Morisky de
adesão a medicação para pacientes com insuficiência cardíaca. Rev Soc Cardiol Estado de São Paulo..

[14] Ciconelli RM, Ferraz MB, Santos W, Meinão I, Marina RQ (1999). Translation to the Portuguese language and general
questionnaire of Quality of Life SF-36 evaluation (Brasil SF-36). Rev Bras Reumatol..

[15] Benetti M, Nahas MV, Barros MVG (2001). Reproducibility and validity of a Brazilian version of
the MacNew quality of life after myocardial infarction (MacNew QLMI)
questionnaire. Med Sci Sports Exerc..

[16] Gollwitzer PM, Brandstatter V (1999). Implementation intentions: Strong effects of simple
plans. American Psychologist.

[17] Orbell S, Sheeran P (2000). Motivational and volitional processes in action
initiation: A field study of implementation intentions. J Appl Soc Psychol..

[18] Rodrigues RCM, Gallani MCBJ, Cornélio ME, Alexandre NMC, São-João TM (2013). The 'Moving Heart Program': an intervention to improve
physical activity among patients with coronary heart disease. Rev. Latino-Am. Enfermagem..

[19] Ventura-Cerdá JM, Mínguez-Gallego C, Fernandez-Villalba EM, Alós-Almiñana M, Andrés-Soler J (2006). Escala simplificada para detectar problemas de
adherencia (ESPA) al tratamiento antirretroviral. Farm Hosp..

[20] Hansen RA, Kim MM, Song L, Tu W, Wu J, Murray MD (2009). Comparison of methods to assess medication adherence and
classify nonadherence. Ann Pharmacother..

[21] Oldridge N, Guyatt G, Jones N, Crowe J, Singer J, Feeny D et al (1991). Effects on quality of life with comprehensive
rehabilitation after acute myocardial infarction. Am J Cardiol..

[22] Valenti L, Lim L, Heller RF, Knapp J (1996). An improved questionnaire for assessing quality of life
after acute myocardial infarction. Qual Life Res..

[23] Nakajima KM, Rodrigues RCM, Gallani MCBJ, Alexandre NMC, Oldridge N (2009). Psychometric properties of MacNew Heart Disease
Health-related Quality of Life Questionnaire: Brazilian version. J Adv Nurs..

[24] Ware JE, Gandek B, IQOLA Project Group (1994). The SF-36 health survey: development and use in mental
health research and the IQOLA Project. Int J Mental Health..

[25] Ajzen I, Fishbein M (1980). Understanding Attitudes and Predicting Social Behavior.

